# A Novel Class of Selective Acetylcholinesterase Inhibitors: Synthesis and Evaluation of (*E*)-2-(Benzo[*d*]thiazol-2-yl)-3-heteroarylacrylonitriles

**DOI:** 10.3390/molecules171012072

**Published:** 2012-10-15

**Authors:** Pedro de la Torre, Luis Astudillo Saavedra, Julio Caballero, Jairo Quiroga, Jans H. Alzate-Morales, Margarita Gutiérrez Cabrera, Jorge Trilleras

**Affiliations:** 1 Laboratorio Síntesis Orgánica, Instituto de Química de Recursos Naturales, Universidad de Talca, Casilla 747, Talca, 3460000, Chile; 2 Centro de Bioinformática y Simulación Molecular, Universidad de Talca, 2 Norte 685, Casilla 721, Talca, 3460000, Chile; 3 Grupo de Investigación de Compuestos Heterocíclicos, Department of Chemistry, Universidad del Valle A. A. 25360 Cali, Colombia; 4 Grupo de Investigación en Compuestos Heterocíclicos, Programa de Química, Facultad de Ciencias Básicas, Universidad del Atlántico, Km 7 Antigua vía Puerto Colombia, A.A.1890, Barranquilla, Colombia

**Keywords:** Knoevenagel condensation, acrylonitriles, AChE inhibitors, docking, ligand-protein interactions, selectivity

## Abstract

(*E*)-2-(benzo[*d*]thiazol-2-yl)-3-heteroarylacrylonitriles are described as a new class of selective inhibitors of acetylcholinesterase (AChE). The most potent compound in the series exhibited good AChE inhibitory activity (IC_50_ = 64 µM). Compound **7f** was found to be more selective than galanthamine in inhibiting AChE and it showed a moderate selectivity index. Kinetic studies on AChE indicated that a competitive type of inhibition pattern exist for these acrylonitrile derivates. Molecular docking models of the ligand-AChE complexes suggest that compound **7g** is located on the periphery of the AChE active site.

## 1. Introduction

**Figure 1:**
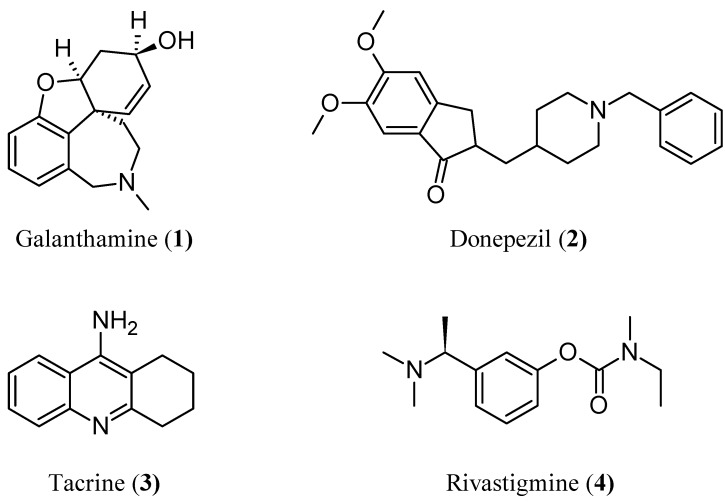
AChE inhibitors currently used in therapeutics to AD.

Alzheimer’s disease (AD) is a progressive neurodegenerative disorder of the central nervous system (CNS) and is one of the most severe health problems of aged people. One promising therapeutic strategy for treating this disease has been the use of acetylcholinesterase (AChE) inhibitors. This enzyme catalyzes the hydrolysis of the neurotransmitter acetylcholine at nerve–nerve synapses and neuromuscular junctions; therefore, its inhibitors lead to the restoration of the levels of acetylcholine. AChE inhibitors such as galanthamine **1** (GAL; Reminyl) [1], donepezil **2** [2], tacrine **3** [3], rivastigmine **4** (Exelon) [4] (Figure 1), and huperzine A (HupA) [5] are the group of drugs most developed and approved for AD symptomatic treatment. Also, in clinical treatment of AD, selective AChE inhibition, has shown better therapeutic effects when compared with nonselective inhibition [6]. Therefore, selectivity of inhibition presents a challenge and an important consideration in any investigation for the development of new types of selective AChE inhibitors because is believed to be an effective approach for AD treatment.

We have previously reported [7] the synthesis of a series of novel (*E*)-2-(benzo[*d*]thiazol-2-yl)-3-arylacrylonitriles. These products were obtained in moderate yields and good purity after short reaction times. In general, in terms of biological activities they are versatile precursors that have been widely used in the synthesis of heterocyclic compounds and as intermediates for the development of new molecules with potential biological or pharmaceutical interest [7,8,9]. Specifically, 2-(benzo[d]- thiazol-2-yl)acetonitrile possess interesting biological properties, including antifungal, antitumor, and antibacterial activities [10,11,12,13,14]. In addition, there are reports that state that compounds containing the acrylonitrile scaffold have antioxidant activity [15], which may have a positive contribution in the treatment of AD [16]. In this work we report the synthesis of a new series of acrylonitrile derivatives characterized by the presence of a 2-(benzo[*d*]thiazol-2-yl)acetonitrile moiety and evaluate them as AChE inhibitors.

## 2. Results and Discussion

### 2.1. Chemistry

A characteristic important of the Knoevenagel adducts of that they are used as intermediates for the development of new molecules with potential biological or pharmaceutical interest, as it is the case of the AChE inhibitors [17].

**Scheme 1:**
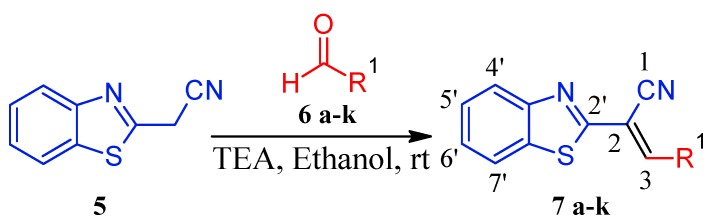
Synthesis of novel (*E*)-2-(benzo[*d*]thiazol-2-yl)-3-hetarylacrylonitriles **7a**–**k**.

In the current work, we reported the synthesis of a new series of (*E*)-2-(benzo[*d*]thiazole-2-yl)-3-heteroarylacrylonitriles **7a**–**k** obtained by Knoevenagel condensation between equimolar amounts of 2-(benzo[*d*]thiazol-2-yl)acetonitrile (**5**) and different substituted hetarylaldehydes **6a**–**k** [18], using ethanol as solvent and catalytic amounts of triethylamine (TEA), after stirring at room temperature for short reaction times (Scheme 1). Adducts were obtained in excellent purities and high yields. The reaction was monitored by Thin Layer Chromatography (TLC) with visualization of the spots by UV light, which evidenced formation of a single product. All the compounds were characterized using NMR spectroscopic techniques, high-resolution mass spectrometry, IR spectroscopy and elemental analyses. ^1^H-NMR analysis of all the derivatives revealed a single olefinic proton, consistent with the formation of a single isomer, which was assigned to have the thermodynamically more stable *E* configuration [18].

**Scheme 2:**
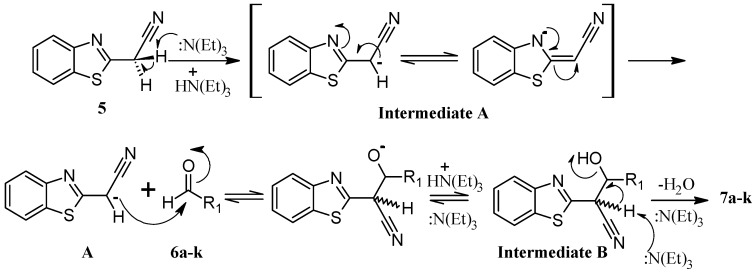
Plausible formation of Knoevenagel adducts in the synthesis of the compounds **7a**–**k**.

It seems reasonable to think that reaction conditions used in this work promote the Knoevenagel reactions through the methylene active component of 2-(benzo[*d*]thiazol-2-yl)acetonitrile (**5**) as a precursor. Under the optimized reaction conditions, in presence of the base TEA, **5** is likely to be converted to intermediate **A**, which contains a nucleophilic carbon susceptible to attack the carbonyl carbon of the aldehydes **6a**–**k** to yield the intermediate **B**, that by elimination of water leads to the expected compounds **7a**–**k** (Scheme 2).

### 2.2. Biological Activities

The biological activity profiles of the compounds **7a**–**k**, as AChE (*Electrophorus electricus*) inhibitors, were assayed in comparison with galanthamine as a reference compound. We also evaluated the inhibitory potency of these compounds against butyrylcholinesterase (BuChE), in order to gain insight about inhibitor’s selectivity against both enzymes. The inhibitory activities were evaluated by the method of Ellman *et al.* [19]. The results are summarized in Table 1. 

**Table 1:** Structures of compounds **7a**–**k** and AChE inhibitory activities.

Entry	R^1^	mp (°C)	Yield (%)	Time reaction (min)	IC_50_^a^ (µM)			Selectivity for AChE
					AChE	BuChE		
**7a**	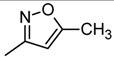	209–211	84	18	1274 ± 0.7	>1870.5 ± 0.6 ^c^		
**7b**	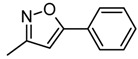	241–243	87	15	1179 ± 0.5	1396.60 ± 0.8		
**7c**	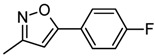	228–230	85	17	699 ± 0.3	>1438.3 ± 1.0 ^c^		
**7d**	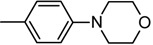	244–246	40	21	504 ± 1.0	>1439.14 ± 1.0 ^c^		
**7e**	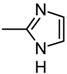	190–192	80	40	303 ± 0.8	>1981.8 ± 0.9 ^c^		
**7f**	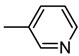	155–157	66	18	103 ± 0.7	>2286.3 ± 0.7 ^c^		22.20
**7g**	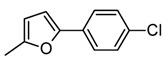	185–187	59	25	64 ± 0.6	474.05 ± 0.8		7.40
**7h**	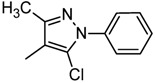	188–190	56	26	>1326.7 ±0.7 ^c^		>1326.75 ± 1.0 ^c^	
**7i**	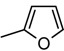	138–140	51	22	>1981.80 ± 1.0 ^c^		1883 ± 0.8	
**7j**	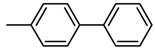	191–193	84	20	>1477.45 ± 1.0 ^c^		1273 ± 0.7	
**7k**	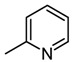	158–160	83	14	>1898.90 ± 0.8 ^c ^		>1899 ± 0.8 ^c^	
Galanthamine					0.54 ± 0.7		8.80 ± 0.5	16.29

^a^ Assay performed using AChE from *Electrophorus electricus *and equine serum. Values are the average from three independent experiments. ^b^ Selectivity for AChE is defined as IC_50_ (BuChE)/IC_50_ (AChE). ^c^ The value of IC_50_ against AChE or BuChE is greater than 0.5 mg/mL. For **7f **the IC_50_ = 0.602 mg/mL. ^d^ Values in the literature [1,20] are 0.36–0.61 µM.

### 2.3. Kinetic Study

**Figure 2:**
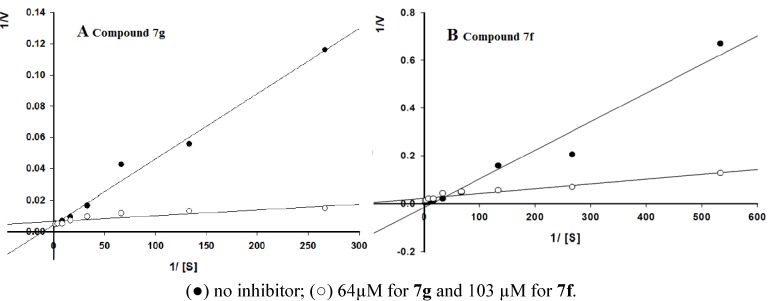
Lineweaver-Burk plot of AChE (0.02U) with substrate acetylthiocholine, in the absence and presence of inhibitors **7g** (**A**), **7f** (**B**).

The kinetics of this new class of AChE inhibitors were studied in detail using compounds **7f** and **7g** as examples. The nature of AChE inhibition, caused by these two compounds, was investigated by the graphical analysis of steady-state inhibition data (Figure 2A,B). Reciprocal plots (Lineweaver-Burk plots) described compounds **7g** and **7f** as competitive inhibitors. The values of *K*_m_ and *V*_max_ were calculated by nonlinear regression according to studies by Abell *et al.* [17], where* V*_max_ was maintained at the same value while *K*_m_ value was increased. For compound **7g** the *K*_m_ and *V*_max_ values were 24.39 and 0.2236, respectively, meanwhile for compound **7f** were 26.36 and 0.07078 respectively.

Compounds **7h**–**k** showed poor inhibition against AChE. The more active compound **7g** (IC_50_ = 64 µM) contains a [5-(4-chlorophenyl)furan-2-yl] substituent. The presence of other substituents instead of furan-2-yl such as 3-isoxazolyl (**7b**; IC_50_ = 1179 µM), phenyl (**7d**; IC_50_ = 504 µM), 1*H*-imidazol-2-yl (**7e**; IC_50_ = 303 µM), and pyridinyl (**7f**; IC_50_ = 103 µM) decreased the AChE inhibitory activity. Furthermore, when the 4-chlorophenyl group, at position 5 of furan-2-yl substituent in compound **7g**, is removed (compound **7i**) the activity also decreases (IC_50_ = 1982 µM). This suggests that chlorophenyl group in **7g** is accommodated along the gorge of AChE. *In vitro* BuChE inhibition was also determined using the same method and all compounds that were tested showed poor inhibition against BuChE. The compound **7g** was also the most active compound against BuChE (IC_50_ = 474 µM) with a moderate selectivity for AChE over BuChE (selectivity index = 7.40). Interestingly, the compound **7f** (IC_50_ = 155 µM against AChE) has a better selectivity index (22.20) for AChE over BuChE. The selectivity toward AChE is interesting for these two compounds when comparing with the selectivity of galanthamine (selectivity index = 16.29).

### 2.4. Molecular Docking

With the aim to understand the ligand–protein interactions at atomic level detail, molecular docking studies were performed for the most potent AChE inhibitor reported in this work (compound **7g**). Compound **7g** is a long chain molecule and we expected that its binding mode, within the AChE binding site, would share some common characteristics with the AChE–donepezil complex.

**Figure 3:**
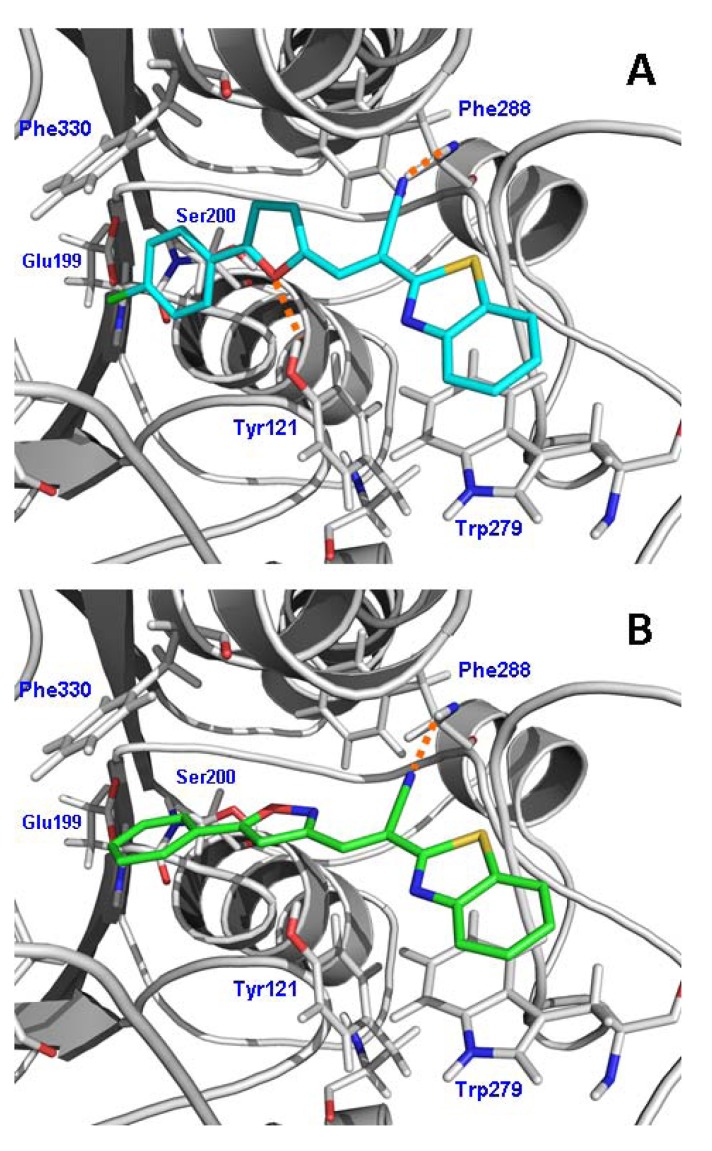
Molecular docking models for compounds **7g** (**A**) and **7b** (**B**) within the AChE binding site highlighting the protein residues that form the main interactions with the inhibitors.

As can be seen in Figure 3A, compound **7g** has several interactions along the active-site gorge of AChE. At the top of the gorge, the phenyl ring of the benzo[*d*]thiazol-2-yl group stacks against the Trp279 indole ring through a π–π interaction. In the middle of the gorge, the nitrogen atom from nitrile group forms a hydrogen bond with the backbone nitrogen of the Phe288. Near the bottom of the gorge, the oxygen atom from furanyl group forms a hydrogen bond with the hydroxyl group of the Tyr121, while the Cl-phenyl substituent at the furanyl group is located at the bottom of the gorge close to Phe330. According to our docking results, (*E*)-2-(benzo[*d*]thiazol-2-yl)-3-heteroarylacrylonitrile derivatives form interactions in accordance with those previously described for donepezil and other AChE inhibitors [2,21].

Compound **7g** is the best inhibitor in our reported series. The main structural difference of this compound with respect to the others is the presence of the 5-(4-chlorophenyl)-2-furyl group at position 3 of acrylonitrile. We also obtained a docking model for the complex between AChE and compound **7b**, in order to explain why the substitution of a furanyl group by an isoxazolyl group decreases the activity. As can be seen in Figure 3B, compound **7b** established almost all the interactions mentioned above, but the isoxazolyl group did not establish hydrogen bond interactions with Tyr121. In this sense, we observed that the 5-(4-chlorophenyl)-2-furyl substituent occupies the site at the bottom of the gorge (by means of the 4-chlorophenyl group) and forms an additional hydrogen bond (by means of the furyl substituent), which has an important contribution to the binding potency of the compound **7g**.

In general, all reported compounds exhibited selectivity for AChE over BuChE. This seems to be due to an appropriate orientation adopted by the benzo[*d*]thiazole scaffold in its binding to the peripheral site of AChE, via the π–π stacking interaction with Trp279 (Figure 3). In BuChE, the peripheral site is surrounded with aliphatic amino acids instead of the aromatic residues present in AChE (Ala277 instead of Trp279). Thus, the π–π stacking interaction observed in AChE cannot be established in BuChE.

## 3. Experimental

### 3.1. General

All solvents used were of analytical grade. Melting points were recorded on a Buchi apparatus and are uncorrected, IR spectra, KBr pellets, 500–4000 cm^−1^ were recorded on a Thermo Nicolet NEXUS 670 FT-IR spectrophotometer, with 0.125 cm^−1^ spectral resolution. ^1^H-NMR and ^13^C-NMR spectra were recorded on a Bruker AM 400 instrument. Chemical shifts are expressed as values relative to TMS as internal standard. Multiplicities are designated as singlet (s), doublet (d), triplet (t), quadruplet (q), multiplet (m). High-resolution mass spectrometry ESI-MS and ESI-MS/MS analyses were conducted in a high-resolution hybrid quadrupole (Q) and orthogonal time-of-flight (TOF) mass spectrometer (Waters/Micromass Q-TOF micro, Manchester, UK) with a constant nebulizer temperature of 100 °C. The experiments were carried out in positive ion mode, and the cone and extractor potentials were set at 10 and 3.0 V, respectively, with a scan range of *m/z* 100–600. MS/MS experiments were carried out by mass selection of a specific ion in Q1, which was then submitted to collision-induced dissociation (CID) with helium in the collision chamber. The product ion MS analysis was accomplished with the high-resolution orthogonal TOF analyzer. The samples were directly infused into the ESI source, via a syringe pump, at flow rates of 5 µL min^−1^, via the instrument’s injection valve. The elemental analyses have been obtained using a LECO CHNS-900 and Thermo Finnigan FlashEA1112 CHNS-O (STIUJA) elemental analyzers. TLC was done on pre-coated silica gel 60 F254 plates (Merck).

*General procedure for the preparation of the (E)-2-(benzo[d]thiazol-2-yl)-3-arylacrylonitriles* **7a**–**k**. A mixture of 2-(benzo[*d*]thiazol-2-yl)acetonitrile (**5**, 1 mmol) and commercial aromatic aldehydes **6a**–**k** with electron donating/withdrawing groups (1 mmol) were reacted with stirring at room temperature for 3 to 10 min using ethanol (10 mL) as solvent and catalytic amounts of triethylamine (0.2 mL). The reactions were monitored by TLC, in order to verify the consumption of the precursors. The solid products were isolated by simple filtration and washed with a mixture of hexane/ethanol (7:3) to give the corresponding compounds.

*(E)-2-(Benzo*[*d*]*thiazol-2-yl)-3-(5-methylisoxazol-3-yl)acrylonitrile * (**7a**). White solid. Yield 84%. mp 209–201 °C. IR (cm^−1^): 3236, 3072, 2991, 2227 (CN), 1596, 1429, 1312, 893, 762. ^1^H-NMR (CDCl_3_): δ ppm 8.19 (s, 1H, H-C=), 8.14 (d, 1H, *J* = 8.07 Hz, H4′_BT_), 7.93 (d, 1H, *J* = 7.58 Hz, H7′_BT_), 7.55 (m, 1H, H6′_BT_), 7.48 (m, 1H, H5′_BT_), 7.02 (s, 1H, CH_isoxazole_), 2.54 (s, 3H, CH_3_); ^13^C-NMR (CDCl_3_): δ ppm 171.95 (C5_isoxazole_), 161.20 (C2′_BT_), 158.40 (C3_isoxazole_), 153.96 (C8′_BT_), 135.53 (C3), 134.84 (C9′_BT_), 127.63 (C5′_BT_), 127.20 (C6′_BT_), 124.69 (C4′_BT_), 122.16 (C1_CN_), 115.33 (C7′_BT_), 112.34 (C4_isoxazole_), 100.97 (C2), 12.83 (CH_3_); EI-MSMS (*m/z*): 267 (M^+^, 100), 252 (30), 226 (46), 199 (20). Anal. Calcd for C_14_H_9_N_3_OS: C, 62.91; H, 3.39; N, 15.72. Found: C, 62.72; H, 3.66; N, 15.83.

*(E)-2-(Benzo[d]thiazol-2-yl)-3-(5-phenylisoxazol-3-yl)acrylonitrile * (**7b**). Green solid. Yield 87%. mp 241–243 °C. IR (cm^−1^): 3066, 3060, 2912, 2220 (CN), 1570, 1450, 1310, 943, 756. ^1^H-NMR (CDCl_3_): δ ppm 8.39 (s, 1H, H-C=), 8.26 (d, 1H, *J *=7.09 Hz, H4′_BT_), 8.17 (d, 1H, *J * = 7.83 Hz, H7′_BT_), 7.99 (m, 2H, H*_o_*), 7.66 (m, 1H, H6′_BT_), 7.60–7.58 (m, 4H, H5′, H*_m_*, H*_p_*), 7.56 (s, 1H, CH_isoxazolyl_); ^13^C-NMR (CDCl_3_): δ ppm, 163.52 (C5_isoxazole_), 161.40 (C2′_BT_), 159.33 (C3_isoxazole_), 155.45 (C8′_BT_), 137.07 (C9′_BT_), 130.05 (C*_p_*), 129.42 (C*_i_*), 128.15 (C*_m_*), 127.99 (C5′_BT_), 126.15 (C*_o_*), 126.09 (C6′_BT_), 122.25 (C3), 121.80 (C4′_BT_), 121.22 (C2), 120.95 (C7′_BT_), 119.32 (C1_CN_), 100.02 (C4_isoxazole_); EI-MS (*m/z*): 329 (M^+^, 30), 318 (50), 302 (20), 274 (100), 171 (8), 218 (10). Anal. Calcd for C_19_H_11_N_3_OS: C, 69.28; H, 3.37; N, 12.76. Found: C, 69.33; H, 3.27; N, 12.98.

*(E)-2-(Benzo[d]thiazol-2-yl)-3-*[5-(4-fluorophenyl)isoxazol-3-yl]*acrylonitrile* (**7c**). White solid. Yield 85%. mp 228–230 °C. IR (cm^−1^) 3127, 3018, 2229 (CN), 1602, 1500, 1438, 1165, 948, 841. ^1^H-NMR (DMSO-*d_6_*): δ ppm 8.39 (s, 1H, H-C=), 8.26 (d, 1H, *J* = 7.58 Hz, H4′_BT_), 8.17 (d, 1H, *J *= 7.34 Hz, H7′_BT_), 8.06 (d, 2H, H*_o_*), 7.65–7.58 (m, 2H, H6′_BT_ and H7′_BT_), 7.55 (s, 1H, CH_isoxazole_), 7.45 (d, 2H, H*_m_*); ^13^C-NMR (CDCl_3_): δ ppm 163.47 (C5_isoxazole_), 161.34 (C2′_BT_), 159.35 (C3_isoxazole_), 158.99 (C*_p_*), 155.43 (C8′_BT_), 137.05 (C9′_BT_), 127.99 (C5′_BT_), 127.55 (C*_o_*), 126.09 (C6′_BT_), 125.19 (C*_i_*), 122.25 (C_3_), 121.90 (C4′_BT_), 121.04 (C_2_), 120.90 (C7′_BT_), 119.25 (C1_CN_), 116.39 (C*_m_*), 100.05 (C4_isoxazole_); EI-MS (*m/z*): 347 (M^+^, 13), 330 (8), 318 (18), 302 (10), 274 (28), 217 (7), 187 (12), 162 (25), 168 (100). Anal. Calcd for C_19_H_10_FN_3_OS: C, 65.70; H, 2.90; N, 12.10. Found: C, 65.92; H, 2.85; N, 12.50.

*(E)-2-(Benzo[d]thiazol-2-yl)-3-(4-morpholin-4-ylphenyl)acrylonitrile * (**7d**). Orange solid. Yield 40%. mp 244–246 °C. IR (cm^−1^): 3053, 2959, 2843, 2214 (CN), 1575, 1509, 1199, 1116. ^1^H-NMR (CDCl_3_): δ ppm 8.12 (s, 1H, H-C=), 8.04 (d, 1H, *J *= 8.0 Hz, H4′_BT_), 8.00 (d, 2H, *J *= 9.05 Hz, H*_o_*), 7.89 (d, 1H, *J* = 7.83 Hz, H7′_BT_), 7.52 (m, 1H, H6′_BT_), 7.39 (m, 1H, H5′_BT_), 6.93 (d, 2H, *J = *9.05 Hz, H*_m_*), 3.88 (m, 4H, =N-CH_2-morpholine_), 3.37 (m, 4H, -O-CH_2-morpholine_); ^13^C-NMR (CDCl_3_): δ ppm 153.74 (C2′_BT_), 153.44 (C8′_BT_), 146.49 (C3), 134.73 (C9′_BT_), 133.13 (C*_p_*), 132.77 (C*_o_*), 126.70 (C5′_BT_), 125.40 (C6′_BT_), 123.09 (C4′_BT_), 122.78 (C*_i_*), 121.55 (C1_CN_), 117.66 (C7′_BT_), 113.86 (C*_m_*), 100.07 (C2), 66.48 (C2_morpholine_ and C6_morpholine_), 47.15 (C3_morpholine_ and C5_morpholine_); EI-MS (*m/z*): 347 (M^+^, 13), 321 (13), 262 (7), 226 (50), 210 (100), 191 (12). Anal. Calcd for C_20_H_17_N_3_OS: C, 69.14; H, 4.93; N, 12.09. Found: C, 69.01; H, 4.94; N, 12.01.

*(E)-2-(Benzo[d]thiazol-2-yl)-3-(1H-imidazol-2-yl)acrylonitrile * (**7e**). White solid. Yield 80%. mp 190–192 °C. IR (cm^−1^): 2930, 2223 (CN), 1436, 1302, 1089, 942, 754; ^1^H-NMR (CDCl_3_): δ ppm 10.74 (s, 1H, NH), 8.10 (s, 1H, H-C=), 8.08 (d, 1H, H4′_BT_), 7.90 (d, 1H, *J *= 7.82 Hz, H7′_BT_), 7.53 (m, 1H, H6′_BT_), 7.48–7.36 (m, 3H, H7′_BT_, H4_imidazole_ and H5_imidazole_); ^13^C-NMR (CDCl_3_): δ ppm 166.06 (C2′_BT_), 154.68 (C8′_BT_), 154.29 (C2_imidazole_), 135.22 (C9′_BT_), 133.66 (C3), 127.46 (C5′_BT_), 126.84 (C6′_BT_), 124.32 (C5_imidazole_), 122.71 (C1_CN_), 122.05 (C4′_BT_), 120.90 (C7′_BT_), 120.21 (C4_imidazole_), 117.41 (C2); EI-MS (*m/z*): 252 (M^+^, 100), 226 (18), 174 (8). Anal. Calcd for C_13_H_8_N_4_S: C, 61.89; H, 3.20; N, 22.21. Found: C, 61.70; H, 2.99; N, 22.10.

*(E)-2-(Benzo[d]thiazol-2-yl)-3-(pyridin-3-yl)acrylonitrile * (**7f**). Green solid. Yield 66%. mp 155–157 °C. IR (cm^−1^): 3055, 2989, 2215 (CN), 1551, 1465, 1437, 1313, 1189, 983; ^1^H-NMR (DMSO-*d_6_*): δ ppm 9.14 (d, 1H, *J* = 2.20 Hz, H2_pyridinyl_), 8.75 (dd, 1H, *J*_1_ = 1.47 Hz, *J*_2_ = 4.89 Hz, H4_pyridinyl_), 8.52 (m, 1H, H5_pyridinyl_), 8.51 (s, 1H, H-C=), 8.23 (dd, 1H, *J*_1_ = 7.95 Hz, *J*_2_ = 0.61 Hz, H4′_BT_), 8.12 (d, 1H, *J* = 7.58 Hz, H7′_BT_), 7.66 (dd, 1H, *J*_1_ = 8.31 Hz, *J*_2_ = 4.89 Hz, H6_pyridinyl_), 7.61 (m, 1H, H6′_BT_), 7.54 (m, 1H, H5′_BT_); ^13^C-NMR (DMSO-d_6_): δ ppm 163.03 (C2′_BT_), 153.17 (C8′_BT_), 152.61 (C4_pyridinyl_), 151.81 (C6_pyridinyl_), 145.68 (C2_pyridinyl_), 136.36 (C9′_BT_), 134.80 (C3), 128.97 (C*_i_*), 127.64 (C5′_BT_), 126,97 (C6′_BT_), 124.46 (C4′_BT_), 123,70 (C5_pyridinyl_), 122.98 (C1_CN_), 116.10 (C7′_BT_), 108.13 (C2); EI-MS (*m/z*): 263 (M^+^, 100), 226 (20), 210 (38). Anal. Calcd for C_15_H_9_N_3_S: C, 68.42; H, 3.45; N, 15.96. Found: C, 68.20; H, 3.70; N, 15.86.

*(E)-2-(Benzo[d]thiazol-2-yl)-3-*[5-(4-chlorophenyl)furan-2-yl]*acrylonitrile * (**7g**). Yellow solid. Yield 59%. mp 185–187 °C. IR (cm^−1^): 3122, 3054, 2916, 2211 (CN), 1588, 1463, 109. ^1^H-NMR (CDCl_3_): δ ppm 8.05 (s, 1H, H-C=), 8.02 (d, 1H, H4′_BT_), 7.91 (d, 1H, *J* = 7.58, H7′_BT_), 7.81 (d, 2H, *J* = 8.56 Hz, H*_o_*), 7.51 (m, 1H, *J* = 1.22 Hz, H6′_BT_), 7.45 (d, 2H, *J* = 7.45 Hz, H*_m_*), 7.41 (m, 1H, *J* = 1.22 Hz, H5′_BT_), 7.24 (d, 1H, *J* = 3.67 Hz, H3_furanyl_), 6.90 (d, 1H, *J* = 3.67 Hz, H4_furanyl_); ^13^C-NMR (CDCl_3_): δ ppm 162.56 (C2′_BT_), 157.64 (C9′_BT_), 153.78 (C5_furanyl_), 148.63 (C2_furanyl_), 135.37 (C8′_BT_), 135.17 (C*_p_*), 130.33 (C*_i_*), 129.41 (C*_m_*), 127.48 (C3), 126.92 (C5′_BT_), 126.35 (C*_o_*), 125.71 (C6′_BT_), 123.24 (C3_furanyl_), 122.72 (C4′_BT_), 121.68 (C1_CN_), 116.89 (C7′_BT_), 109.15 (C4_furanyl_), 100.54 (C2); EI-MS (*m/z*): 363 (M^+^, 8), 321 (13), 261 (8), 210 (100), 193 (42), 152 (27), 115 (20). Anal. Calcd for C_20_H_11_ClN_2_OS: C, 66.21; H, 3.06; N, 7.72. Found: C, 66.10; H, 3.12; N, 7.55.

*(E)-2-(Benzo[d]thiazol-2-yl)-3-(5-chloro-3-methyl-1-phenyl-1H-pyrazol-4-yl)acrylonitrile * (**7h**). Green solid. Yield 59%. mp 188–190 °C. IR (cm^−1^): 3053, 2917, 2213 (CN), 1589, 1510, 1409, 1309, 896; ^1^H-NMR (CDCl_3_): δ ppm 8.14 (s, 1H, H-C=), 8.10 (d, 1H, *J* = 8.07 Hz, H4′_BT_), 7.92 (d, 1H, *J* = 7.58 Hz, H7′_BT_), 7.60 (m, 1H, H6′_BT_), 7.54 (m, 1H, H5′_BT_); 7.60–7.42 (m, 5H, H*_o,p,m_*_-fenyl_), 2.56 (s, 3H, CH_3_); ^13^C-NMR (CDCl_3_): δ ppm 162.20 (C2′_BT_), 153.63 (C9′_BT_), 149.88 (C5_pyrazole_), 137.43 (C3_pyrazole_), 136.81 (C*_i_*), 135.07 (C8′_BT_), 129.32 (C*_p_*), 129.23 (C*_m_*), 128.97 (C3), 126.97 (C5′_BT_), 126.03 (C6′_BT_), 124.99 (C*_o_*), 123.63 (C4′_BT_), 121.69 (C1_CN_), 116.28 (C7′_BT_), 113.67 (C4_pyrazole_), 107.93 (C2), 15.05 (CH3); EI-MS (*m/z*): 377 (M^+^, 9), 341 (19), 340 (15), 274 (23), 246 (45), 205 (22), 168 (100), 135 (22), 102 (90). Anal. Calcd for C_20_H_13_ClN_4_S: C, 63.74; H, 3.48; N, 14.87. Found: C, 63.71; H, 3.51; N, 14.83.

*(E)-2-(Benzo[d]thiazol-2-yl)-3-(2-furyl)acrylonitrile * (**7i**). Yellow solid. Yield 51%. mp 138–140 °C. IR (cm^−1^): 3120, 3035, 2914, 2214 (CN), 1607, 1452, 1305, 882; ^1^H-NMR (CDCl_3_): δ ppm 8.07 (s, 1H, H-C=), 8.05 (d, 1H, *J* = 8.07 Hz, H4′_BT_), 7.90 (d, 1H, *J* = 8.07 Hz, H7′_BT_), 7.73 (d, 1H, *J* = 1.47 Hz, H5_furanyl_), 7.52 (m, 1H, H6′_BT_), 7.41 (m, 1H, H5′_BT_), 7.32 (d, 1H, *J* = 3.67 Hz, H3_furanyl_), 6.65 (dd, 1H, *J_1_* = 3.55, *J_2_* = 1.59 Hz, H4_furanyl_); ^13^C-NMR (CDCl_3_) δ ppm 162.28 (C2′_BT_), 153.69 (C9′_BT_), 149.27 (C2_furanyl_), 147.15 (C5_furanyl_), 135.08 (C8′_BT_), 131.69 (C3_furanyl_), 126.92 (C3), 125.82 (C5′_BT_), 123.38 (C6′_BT_), 121.67 (C4′_BT_), 119.66 (C1_CN_), 116.35 (C7′_BT_), 113.63 (C4_furanyl_), 101.44 (C2); EI-MS (*m/z*): 252 (23), 134 (8), 124 (70), 110 (52), 102 (100). Anal. Calcd for C_14_H_8_N_2_OS: C, 66.65; H, 3.20; N, 11.10. Found: C, 66.62; H, 3.10; N, 10.99.

*(E)-2-(Benzo[d]thiazol-2-yl)-3-(1,1'-biphenyl-4-yl)acrylonitrile * (**7j**). Green solid. Yield 84%. mp 191–193 °C. IR (cm^−1^): 3057, 3011, 2216 (CN), 1583, 1480, 1302; ^1^H-NMR (CDCl_3_): δ ppm 8.28 (s, 1H, H-C=), 8.12 (d, 2H, *J* = 8.55 Hz, H*_o_*), 8.08 (d, 1H, H4′_BT_), 7.92 (d, 1H, *J* = 7.58 Hz, H7′_BT_), 7.76 (d, 2H, *J* = 8.56 Hz, H*_m_*), 7.66 (d, 2H, *J* = 7.34 Hz, H′*_o_*), 7.54 (m, 1H, H6′_BT_), 7.50–7.39 (m, 4H, H′*_m_*, H′*_p_* and H5′_BT_); ^13^C-NMR (CDCl_3_): δ ppm 162.86 (C2′_BT_), 153.63 (C9′_BT_), 146.33 (C3), 144.94 (C1_CN_), 139.56 (C′*_i_*), 134.98 (C8′_BT_), 131.26 (C*_i_*), 130.98 (C*_o_*)*,* 129.03 (C′*_o_*), 128.39 (C′*_p_*), 127.77 (C′*_m_*), 127.16 (C*_m_*), 126.95 (C5′_BT_), 125.98 (C6′_BT_), 123.57 (C4′_BT_), 121.68 (C1_CN_), 116.66 (C7′_BT_), 105.08 (C2); EI-MS (*m/z*): 338 (M^+^, 5), 246 (20), 203 (7), 162 (6), 124 (100), 101 (41). Anal. Calcd for C_22_H_14_N_2_S: C, 78.08; H, 4.17; N, 8.28. Found: C, 78.05; H, 4.14; N, 8.27.

*(E)-2-(Benzo[d]thiazol-2-yl)-3-(pyridin-2-yl)acrylonitrile* (**7k**). Green solid. Yield 83%. mp 158–160 °C. IR (cm^−1^): 3052, 2992, 2217(CN), 1557, 1469, 1433, 1313, 1171, 980; ^1^H-NMR (CDCl_3_): δ ppm 8.84 (d, 1H, *J* = 4.65 Hz, H3_pyridinyl_), 8.29 (s, 1H, H-C=), 8.12 (d, 1H, *J* = 8.07 Hz, H4′_BT_), 7.94 (d, 1H, *J* = 7.58 Hz, H7′_BT_), 7.89 (d, 1H, H6_pyridinyl_), 7.84 (m, 1H, H5_pyridinyl_), 7.55 (m, 1H, H6′_BT_), 7.46 (m, 1H, H5′_BT_), 7.40 (dd, 1H, H4_pyridinyl_); ^13^C-NMR (CDCl_3_): δ ppm 162.89 (C2′_BT_), 154.05 (C8′_BT_), 151.20 (C4_pyridinyl_), 150.81 (C6_pyridinyl_), 145.30 (C3_pyridinyl_), 137.38 (C9′_BT_), 135.75 (C3), 127.45 (C*_i_*), 126.71 (C5′_BT_), 126.43 (C6′_BT_), 125.85 (C4′_BT_), 124.28 (C5_pyridinyl_), 122.18 (C1_CN_), 116.44 (C7′_BT_), 109.43 (C2); EI-MS (*m/z*): 263 (M^+^,100), 262 (70), 200 (37), 180 (20), 152 (33), 185 (4). Anal. Calcd for C_15_H_9_N_3_S: C, 68.42; H, 3.45; N, 15.96. Found: C, 68.40; H, 3.41; N, 15.93.

### 3.2. Pharmacology

#### 3.2.1. AChE Assay

In 96-well plates, the sample (50 μL) was dissolved in phosphate buffer (8 mM K_2_HPO_4_, 2.3 mM NaH_2_PO_4_, 150 mM NaCl, and 0.05% Tween 20 at pH 7.6) and an AChE/BuChE solution (50 μL, 0.25 unit/mL) from *Electroporus electricus* and equine serum, respectively, in the same phosphate buffer, were added. The assay solutions, except substrate, were preincubated with the enzyme for 30 min at room temperature. After preincubation, the substrate was added. The substrate solution consists of Na_2_HPO_4_ (40 mM), acetylthiocholine/butyrylthiocholine (0.24 mM) and 5,5′-dithio-bis-(2-nitrobenzoic acid) (0.2 mM, DTNB, Ellman’s reagent). Absorbance of the yellow anion product, due to the spontaneous hydrolysis of substrate, was measured at 405 nm for 5 min on a Microtiter plate reader (Multiskan EX, Thermo, Vantaa, Finland). The AChE/BuChE inhibition was determined for each compound. The enzyme activity was calculated as a percentage compared to a control using only the buffer and enzyme solution. The compounds were assayed in the dilution interval of 500 to 15 μg/mL, and the alkaloid galanthamine was used as the reference compound. Each assay was run in triplicate and each reaction was repeated at least three independent times. The IC_50_ values were calculated by means of regression analysis. The IC_50_ value of galanthamine was developed in three plates in a range 250 μg/mL to 0.122 μg/mL, in the first plate the value was 0.179 μg/mL (0.486 µM), in the second was 0.213 μg/mL (0.578 µM), and the third was 0.193 μg/mL (0.524 µM), which gives an average value of 0.54 μM.

#### 3.2.2. Kinetic Characterization of AChE Inhibition

Kinetic characterization of AChE was performed by means of Ellman’s method. The effect of inhibitor **7g** and **7f** on varying concentration of substrate from 0.48 to 9.38 × 10^−4^ mM was investigated. Enzyme kinetic characterization studies were performed under same incubation conditions as described above using acetylthiocholine as substrate and DTNB was used as chromophoric reagent. A parallel control with no inhibitor in the mixture was used for comparison. Each concentration was analyzed in triplicate; and Lineweaver-Burk (1/V *vs.* 1/[S]) plot was constructed using Sigma Plot 10.0 version software [22].

#### 3.2.3. Molecular Docking

Docking was used to suggest the binding modes of some of the reported inhibitors. Docking was performed using Glide [23]. The Glide program is contained in Maestro 9.0 software [24]. Glide docking uses a series of hierarchical filters to find the best possible ligand binding locations in a previously built receptor grid space. The filters include a systematic search approach, which samples the positional, conformational, and orientational space of the ligand before evaluating the energy interactions between the ligand and the protein [23].

The protein coordinates were extracted from the X-ray crystal structure of the AChE–donepezil complex [accession code in the Protein Data Bank (PDB): 1EVE]. The structures of the compounds **7b** and **7g** were sketched with Maestro software. The extra-precision (XP) module of Glide was used. A grid box of 30 Å × 30 Å × 30 Å was first centered on the center of mass of the donepezil in PDB 1EVE. Default docking parameters were used [25]. The docking hierarchy begins with the systematic conformational expansion of the ligand followed by placement in the receptor site. Then minimization of the ligand in the field of the receptor was carried out using the OPLS-AA [26] force field with a distance-dependent dielectric of 2.0. Afterward, the lowest energy poses were subjected to a Monte Carlo procedure that samples the nearby torsional minima. The best pose for a given ligand was determined by the Emodel score, while different compounds were ranked using Glide Score [27]. The docking poses for each ligand were analyzed by examining their relative total energy score. The more energetically favorable conformation was selected as the best pose.

## 4. Conclusions

In summary, a series of (*E*)-2-(benzo[*d*]thiazol-2-yl)-3-heteroarylacrylonitrile derivatives were designed, synthesized, and evaluated as AChE inhibitors. Structure–activity studies showed that AChE inhibitory activity of compounds was influenced by the substituent pattern at position 3 of acrylonitrile. The most potent compound (compound **7g**) contains a 5-(4-chlorophenyl)furan-2-yl group at this position. The molecular modeling study of compound **7g** indicated that it is accommodated inside the active-site gorge of AChE, adopting the same interactions that were described for the AChE–donepezil complex. The results suggest that rational modifications of the substituent in the scaffold (*E*)-2-(benzo[*d*]thiazol-2-yl)-3-(2-furyl)acrylonitrile would provide a rational basis for the development of novel AChE inhibitors. Further studies on this series of derivatives are in progress and will be reported in due course.
